# Sarcoma of the Testicle

**Published:** 1903-08-29

**Authors:** 


### SARCOMA OF THE TESTICLE.

In discussing the nature, diagnosis, and treatment of malignant growths of the testicle, Dr. Coley[1] states that they are much more likely to be sarcomatous than carcinomatous. Of his own eighteen cases, one was a teratoma while the remaining seventeen were all sarcomata. The disease occurs most commonly in young adult life. More than half his own patients were young men between 20 and 30 years old. The youngest patient was 19 and the oldest was 55 years of age. These figures do not bear out the statement of some, that sarcoma of the testis is a disease of childhood and old age. With reference to the etiology, trauma plays an important part. In 50 per cent. of his cases there was a distinct history of antecedent injury, and in most of these the tumour developed so quickly after the receipt of the injury that there could be no doubt about the etiological importance of the latter. Taking sarcomata as a class there is a definite history of antecedent trauma in fully one-third of the cases, while in sarcoma of certain localities this percentage is much higher. In every instance of sarcoma of the breast that Dr. Coley has seen there has been a history of trauma previous to the development of the tumour. In sarcoma of the testis there was a local injury to the testis a short time before the tumour was noticed in nearly one half of the cases. Glandular infection, which either does not take place at all or else only occurs in the latest stages of sarcomata in general, is found very early in cases of sarcoma of the testicle, the inguinal and retro-peritoneal glands being the ones affected. The diagnosis is usually not difficult. The condition for which it is most likely to be mistaken, if any confusion does occur, is tuberculosis, but only rarely is the resemblance close enough to cause a doubt. In one case under Dr. Coley's care tuberculosis of the testicle very closely resembled sarcoma both as regards clinical history and physical characteristics. The disease was of short duration; there was a history of antecedent injury, smooth symmetrical enlargement, no involvement of the skin, no thickening of the cord; a small amount of fluid in the tunica vaginalis; no family history of tuberculosis, and no evidence of tuberculosis elsewhere in the body. The diagnosis of tuberculosis rather than sarcoma in this case was made almost entirely upon the varying consistency of the tumour itself. The prognosis of sarcoma of the testis is far from satisfactory. Kober's statistics show nine cases only out of a total of 105, which remained free from recurrence after operation for from 3 to 15 years. If these nine be considered as cures the percentage of recoveries is only 8·5. The duration of the disease is shorter than sarcoma of other localities. In one of Dr. Coley's cases the course was so rapid that, in spite of castration being performed with removal of the cord as high up as the internal ring four weeks after the first appearance of the tumour, secondary growths occurred in the abdomen and increased in size with extreme rapidity so as to cause the patients' death in a little more than three months from the time the lump in the testicle was discovered. The only rational treatment in every case consists in complete removal of the testis and cord as high up as the internal ring as soon as the diagnosis has been made. The only hope for better results in the future lies in earlier diagnosis and operation, while the practice of delaying for weeks while trying the effect of mercurial ointment so often advocated by text-books and practised by surgeons is, Dr. Coley thinks, to be strongly condemned. Usually a diagnosis can be made in the early stages if sufficient care be taken in the examination, and in cases of doubt, the dangers of delay are so great that an exploratory incision should be made, and the complete operation performed at once if section of the growth confirms the suspicion of sarcoma. In his opinion it is unsafe to remove a portion for microscopical examination, on account of the danger of infected cells being carried to remote parts by the blood current and setting up metastases.

[1] Medical News, July 25.

